# Nano-structural effects on Hematite (α-Fe_2_O_3_) nanoparticle radiofrequency heating

**DOI:** 10.1186/s40580-021-00258-7

**Published:** 2021-03-09

**Authors:** Camilah D. Powell, Amanda W. Lounsbury, Zachary S. Fishman, Christian L. Coonrod, Miranda J. Gallagher, Dino Villagran, Julie B. Zimmerman, Lisa D. Pfefferle, Michael S. Wong

**Affiliations:** 1 Chemical and Biomolecular Engineering, Rice University, Houston, TX USA; 2 Nanosystems Engineering Research Center for Nanotechnology-Enabled Water Treatment, Houston, TX, USA; 3 Civil and Environmental Engineering, Rice University, Houston, TX USA; 4 Chemistry, Rice University, Houston, TX USA; 5 Material Science and NanoEngineering, Rice University, Houston, TX USA; 6 Chemistry, University of Texas At El Paso, El Paso, TX USA; 7 Chemical and Environmental Engineering, Yale University, New Haven, CT USA

**Keywords:** Nanparticles, Magnetism, Hematite, Radiofrequency, Heating

## Abstract

Nano-sized hematite (α-Fe_2_O_3_) is not well suited for magnetic heating via an alternating magnetic field (AMF) because it is not superparamagnetic—at its best, it is weakly ferromagnetic. However, manipulating the magnetic properties of nano-sized hematite (i.e., magnetic saturation (Ms), magnetic remanence (Mr), and coercivity (Hc)) can make them useful for nanomedicine (i.e., magnetic hyperthermia) and nanoelectronics (i.e., data storage). Herein we study the effects of size, shape, and crystallinity on hematite nanoparticles to experimentally determine the most crucial variable leading to enhancing the radio frequency (RF) heating properties. We present the synthesis, characterization, and magnetic behavior to determine the structure–property relationship between hematite nano-magnetism and RF heating. Increasing particle shape anisotropy had the largest effect on the specific adsorption rate (SAR) producing SAR values more than 6 × greater than the nanospheres (i.e., 45.6 ± 3 W/g of α-Fe_2_O_3_ nanorods vs. 6.89 W/g of α-Fe_2_O_3_ nanospheres), indicating α-Fe_2_O_3_ nanorods can be useful for magnetic hyperthermia.

## Introduction

As the most stable iron oxide under acidic [1] and ambient conditions [2], hematite (i.e., α-Fe_2_O_3_) has been heavily studied for a variety of applications including: waste water treatment [2–5], catalysis [6], gas sensors [2], and electrodes [7]. Although environmentally benign and biocompatible [8, 9], bulk hematite is not suitable for radio frequency (RF) magnetic heating applications because it is weakly ferromagnetic at room temperature [10, 11] (i.e., M_S,bulk_ ~ 0.3 emu/g [12]).

An alternating magnetic field (AMF) causes magnetic particles to generate heat by way of four different mechanisms: eddy current heating, hysteretic heating, Brownian relaxation, and Néel relaxation [8]. Bulk size particles (i.e., centimeter scale or larger) undergo eddy current heating which is dependent upon the electrical conductivity/resistivity of a material. For multi-domain nano-sized particles (e.g., ≥ 50 nm), RF heating is generated by domain wall motion (i.e., hysteretic heating) and is closely dependent upon the magnetic saturation of the nanoparticle. For single domain, superparamagnetic-like nanoparticles (e.g., < 50 nm) Brownian and/or Neel relaxations contribute to RF heating [8].

Control of nanoparticle size, shape, and crystallinity of superparamagnetic materials (i.e., Fe_3_O_4_, γ-Fe_2_O_3_, and MFe_2_O_4_ where M = Ni, Mn, or Co [13]) can be manipulated to enhance their magnetic properties and their RF magnetic heating performance making them more suitable for magnetic hyperthermia applications – a promising cancer treatment therapy with encouraging findings for breast carcinoma and brain tumors [14]. However, reasonable concerns surround the toxicity and bioaccumulation of iron oxide nanoparticles within the human body [14, 15]. Hematite nanoparticles can mitigate toxicity issues by serving as an earth-abundant, biocompatible [8, 9] instrument for RF heating. If properly manipulated, nano-scale hematite particles can facilitate heating efficiencies large enough to destroy 4 mm dia. tumors (e.g., ~ 50 W/g [16]) given a clear understanding of the structure property relationships between nanostructure and nano-magnetism. Though studies on the structure–property relationship between hematite nanoparticles and magnetism [17–22] exist, seemingly none – to the authors knowledge – exists for RF heating.

In this work, we synthesized five different hematite samples with different sizes, shapes, and crystallinity to examine the structure–property relationship between hematite nano-magnetism and RF heating. Their wet-chemical syntheses were carried out following procedures previously developed, but their magnetic properties were not characterized [23, 24]. We report that nanostructural changes in hematite nanoparticles elicit marked differences in their magnetic profile and RF heating responses.

## Experimental

### Materials

All materials used for synthesizing the α-Fe_2_O_3_ rugby balls, nanospheres, and nanodiamonds, were ACS grade reagents purchased from Sigma Aldrich. For the α-Fe_2_O_3_ nanosheets and nanorods, all chemicals were purchased from Sigma Aldrich with purity $\ge 97\%$. All gases used were purchased from Airgas at ultra-high purity.

#### Synthesis of α-Fe_2_O_3_ Rugby balls

Rugby ball shaped α-Fe_2_O_3_ particles were synthesized according to procedures outlined in previous works [23]. Succinctly, 500 mL of 0.2 M Fe(ClO_4_)_3_ was rapidly heated to 98 $^\circ$ C and immediately incubated at 98 $^\circ$ C for seven days.

#### Synthesis of α-Fe_2_O_3_ nanospheres

Sphere shaped α-Fe_2_O_3_ nanoparticles were synthesized according to procedures outlined in previous works [23]. In summary, 0.02 M of Fe of fresh FeCl_3_ $\bullet$ 6H_2_O was added to 2 L of 98 $^\circ$ C 0.002 M HCl under stirring. The mixed solution was then sealed and allowed to incubate at 98 $^\circ$ C for 10 days.

#### Synthesis of α-Fe_2_O_3_ nanodiamonds

Diamond shaped α-Fe_2_O_3_ nanoparticles were synthesized according to procedures outlined in previous works [23]. In short, 2.0 L of 0.002 M HNO_3_ was heated to 98 $^\circ$ C. Once heated, 0.02 M Fe of Fe(NO_3_)_3_ · 9H_2_O was added under rapid mixing. The solution was then sealed to incubate for seven days.

#### Synthesis of α-Fe_2_O_3_ nanosheets

Sheets of α-Fe_2_O_3_ nanoparticles were synthesized according to procedures outlined in previous works [24]. In summary, the nanosheets were produced using a hard template of copper oxide (CuO) Nano-Sheets. Upon magnetic stirring, 900 mg of CuO nanosheets were dispersed in 900 mL of DI water; the solution was then heated to 60 $^\circ$ C. 3.4 g of FeSO_4_ · 7H_2_O was dissolved into the heated solution. After 2 h, the solution color changed from black to dark orange. The resulting precipitate was filtered out of the solution and washed with excess DI water. To remove the CuO hard templates, the precipitate was washed three times with 300 mL of concentrated ammonium hydroxide. The remaining orange sample was washed again with DI water, vacuum dried, and crushed into a powder. The crushed powder was then heated in air at 400 $^\circ$ C for 30 min.

#### Synthesis of α-Fe_2_O_3_ nanorods

α-Fe_2_O_3_ nanorods were synthesized according to procedures outlined in previous works [24]. 60 g of NaOH was dissolved into 450 mL of DI water and heated to 50 $^\circ$ C. At the same time, 1.95 g of of FeSO_4_ $\bullet$ 7H_2_O was dissolved into 50 mL of DI water. Nitrogen gas (N_2_) was bubbled through each solution for 30 min to remove dissolved oxygen. Both solutions, the iron sulfate solution and the sodium hydroxide solution, were added together and allowed to react for 1 h under magnetic stirring with N_2_ bubbling at 50 $^\circ$ C. The resulting precipitate was green in color. The precipitate was filtered, washed with excessive DI water under N_2_ gas, vacuum dried, and crushed into a powder. The crushed powder was then heated in air at 400 $^\circ$ C for 15 min.

### Magnetic characterization

Magnetization measurements of all materials were collected with a Superconducting Interference Device (SQUID) complete with a MPMS XL (Quantum Design Inc.). Magnetization curves from -10 kOe to 10 kOe at 300 K were collected for each nanoparticle morphology. For the zero-field cooled (ZFC) and field cooled (FC) curves, magnetic measurements were taken from 5 K to 300 K with a set magnetic field of 100 Oe. For each magnetic measurement the hematite samples were weighed and wrapped in Teflon tape.

### X-ray diffraction (XRD) measurements

Each nanomaterial was analyzed by powder x-ray diffraction (XRD) using a Rigaku SmartLab X-ray diffractometer with Cu K $\alpha$ radiation (1.5418 Å). For each nanomaterial the data was collected from 2θ = 3° to 90°.

### Scanning electron microscopy (SEM)

Dilute dispersions of the hematite samples in ethanol were drop-coated onto silicon wafers and imaged with the Helios 660 SEM/FIB microscope (Thermo Scientific^TM^) with an acceleration voltage of 2.5 or 5 kV.

### Radio frequency (RF) heating experiments

Radio Frequency (RF) Heating experiments were performed using a slightly modified procedure previously used by others [25]*.* A 1 kW EASYHEAT induction heating system (Ambrell), was used to generate an alternating magnetic field at the fixed frequency of 325 kHz. The heating system consisted of a 5-turn coil with peak field strength 13.3 kA/m (5 cm inner dia., 3 cm height).

To measure SAR values, each nanomaterial was dispersed in 1 mL of DI water (i.e., each solution was sonicated for 60 s) and placed inside of a 2 mL cryovial. The cryovial was then insulated with Styrofoam and placed into the center of the inductive coil. The induction heating system was then turned on for 90 s. The temperature of the solution was measured with a real-time fiber optic temperature sensor probe over time (LumaSense Technologies m3300), from which a linear fit of the average slope (dT/dt) of the first 20 s was obtained. The average slope of the solvent alone was subtracted to compensate for heat exchange with the surroundings. SAR was calculated with the following equation below:

1 $${SAR}_{meas}= \frac{1}{{m}_{-{\mathrm{Fe}}_{2}{O}_{3}}}{C}_{sol}{m}_{sol}\left(\frac{dT}{dt}\right)$$

where ${m}_{-{\mathrm{Fe}}_{2}{O}_{3}}$ is the mass of the hematite sample, ${C}_{sol}$ is the specific heat of the solvent (${C}_{{H}_{2}O}=4.184 {\mathrm{JK}}^{-1}{\mathrm{g}}^{-1}$), ${m}_{sol}$ is the mass of the solvent, and $dT/dt$ is the slope of the temperature versus time graph.

## Results and discussion

### Particle characterization

**Fig. 1:**
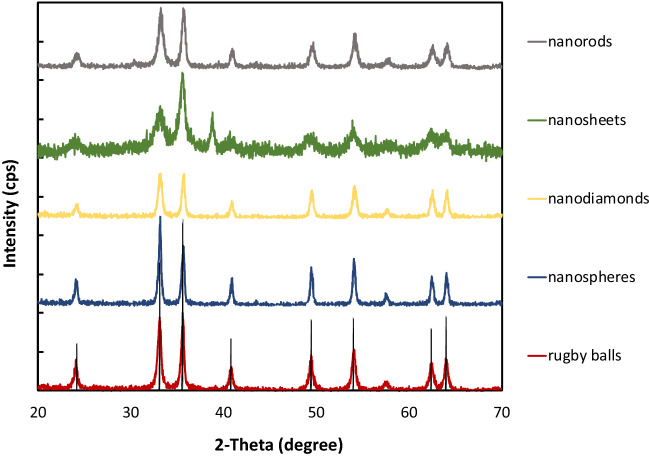
XRD spectra for the hematite nanorods (Grey), nanosheets (Green), nanodiamonds (Yellow), nanospheres (Blue), and rugby balls (Red) shaped particles

**Fig. 2:**
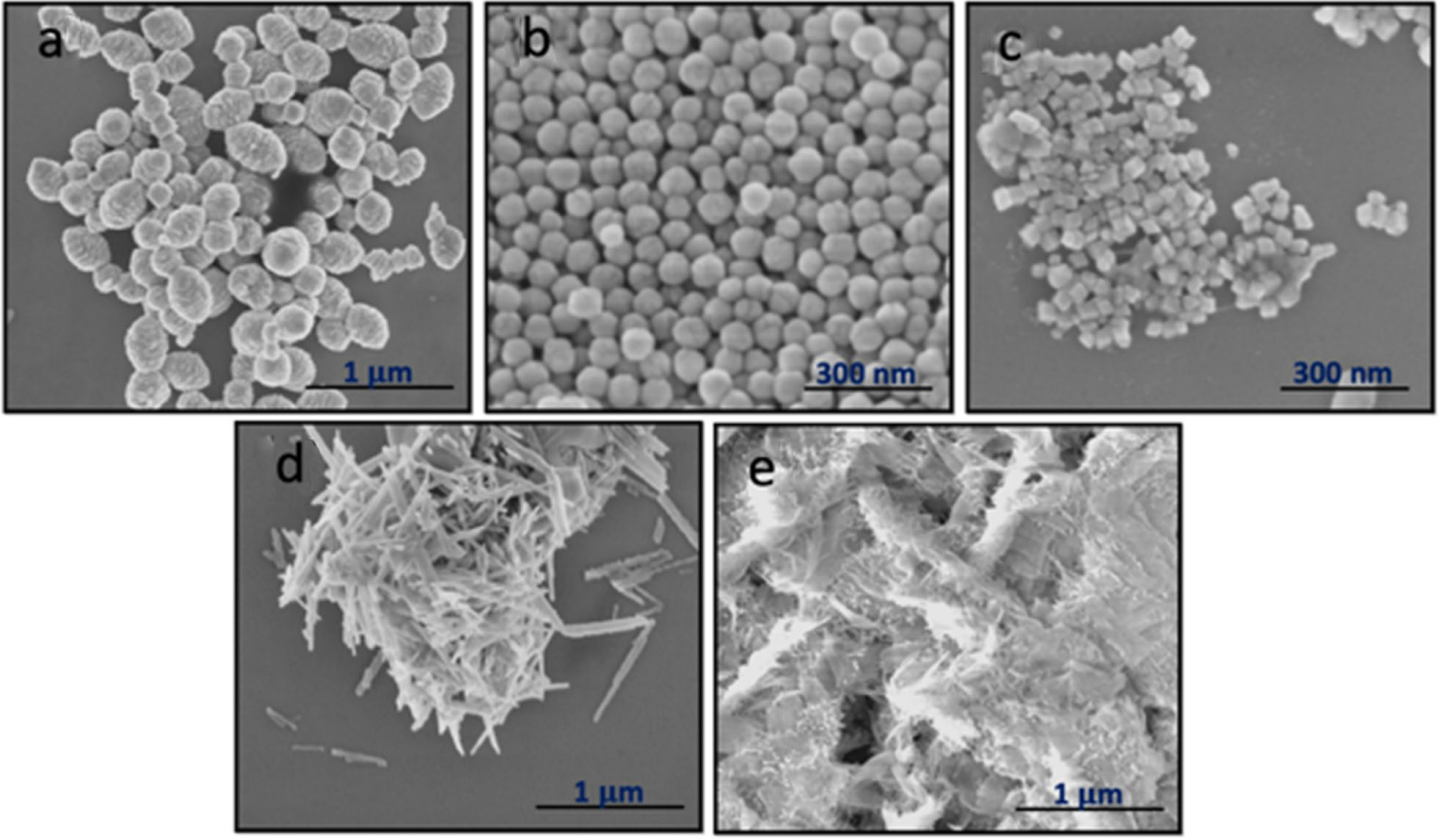
SEM of the α-Fe_2_O_3_ **a** rugby balls, **b** nanospheres, **c** nanodiamonds, **d** nanorods, and **e** nanosheets particles

We confirmed the as-synthesized hematite nanoparticles had the α-Fe_2_O_3_ crystal structure through x-ray diffractometry.^28^ 2θ of 24.2°, 33.2°, 35.6°, 40.9°, and 49.4° corresponding to α-Fe_2_O_3_, are shown in the XRD patterns in Fig. 1. SEM verified the shape, size, and crystallinity of each nanorod, nanodiamond, nanosheet, nanosphere, and rugby ball-shaped sample (Fig. 2, Additional file 1: Figure S1). The sizes of the individual nanoparticles ranged from approx. 4 nm to 150 nm with the nanosheets having the smallest size and with the rugby balls having the largest dimensions (Additional file 1: Figure S2).

### Magnetic characterization

**Fig. 3:**
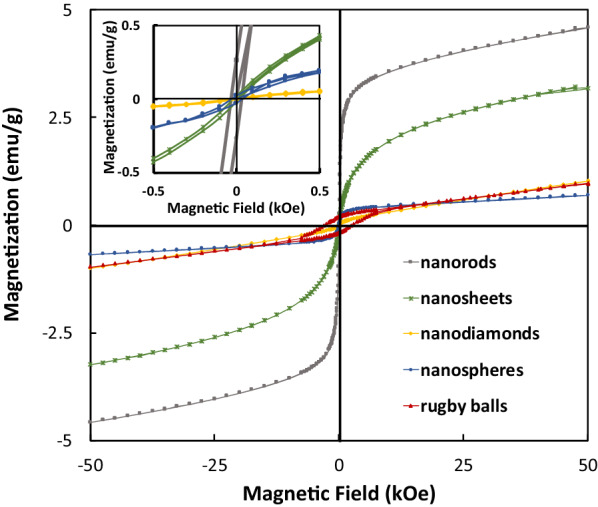
Magnetization curves at 300 K from -50 kOe to -50 kOe for the hematite nanorods (grey squares), nanosheets (green asterisks), nanodiamonds (yellow diamonds), nanospheres (blue spheres), and rugby balls (red triangles) shaped particles

The nanospheres, nanosheets, and nanorods had magnetic saturation (M_s_) values of 0.69 emu/g, 3.25 emu/g, and 4.58 emu/g, respectively (Fig. 3, Table 1). The rugby ball and nanodiamond shaped nanoparticles did not reach magnetic saturation at the maximum magnetic field of 50 kOe. The rugby ball shaped hematite particles (~178 nm) display magnetic behavior similar to that of bulk hematite (~3 µm; weakly ferromagnetic [10–12]) at room temperature [11, 26]*.*

**Table 1:** Sizes and magnetic properties of the hematite shapes

Sample Names	Avg. Size^a^ (nm)	M_s_ (emu/g)	M_r_ (emu/g)	M_r_/M_s_	H_c_ (Oe)
Nanorods	~ 38	4.58	0.264	0.058	30.0
Nanosheets	4–7 × ~ 200 [24]	3.25	0.020	0.006	20.0
Nanodiamonds	~ 24	1.02^b^	0.004	n/a	27.8
Nanospheres	~ 66	0.69	0.020	0.029	27.5
Rugby balls	~ 178	0.96^b^	0.200	n/a	2750

^a^Determined from SEM images

^b^Magnetization value measured at maximum magnetic field (50 kOe)

The other hematite nanoparticle shapes (nanospheres, nanodiamonds, nanosheets, and nanorods) show superparamagnetic-like behavior, based on their very low coercivity (H_c_) and magnetic remanence (M_r_) values [27]. None are technically superparamagnetic, which demands that M_r_/M_s_ ratio = 0. The M_r_/M_s_ ratio (which signifies the extent of ferromagnetism) of the nanospheres and the nanorods were 0.029 and 0.058, respectively, indicating they were ferromagnetic [21]. The hematite nanosheets had a smaller M_r_/M_s_ ratio (= 0.006), indicating that they were closest to acting superparamagnetic. In going from the larger rugby balls (~178 nm) to the smaller nanospheres (~66 nm), the magnetic hysteresis disappears, with magnetic remanence M_r_ (magnetization at zero magnetic field strength) and coercivity H_c_ (magnetic field strength at zero magnetization) values decreasing.

### Zero field and field cooling results

**Fig. 4:**
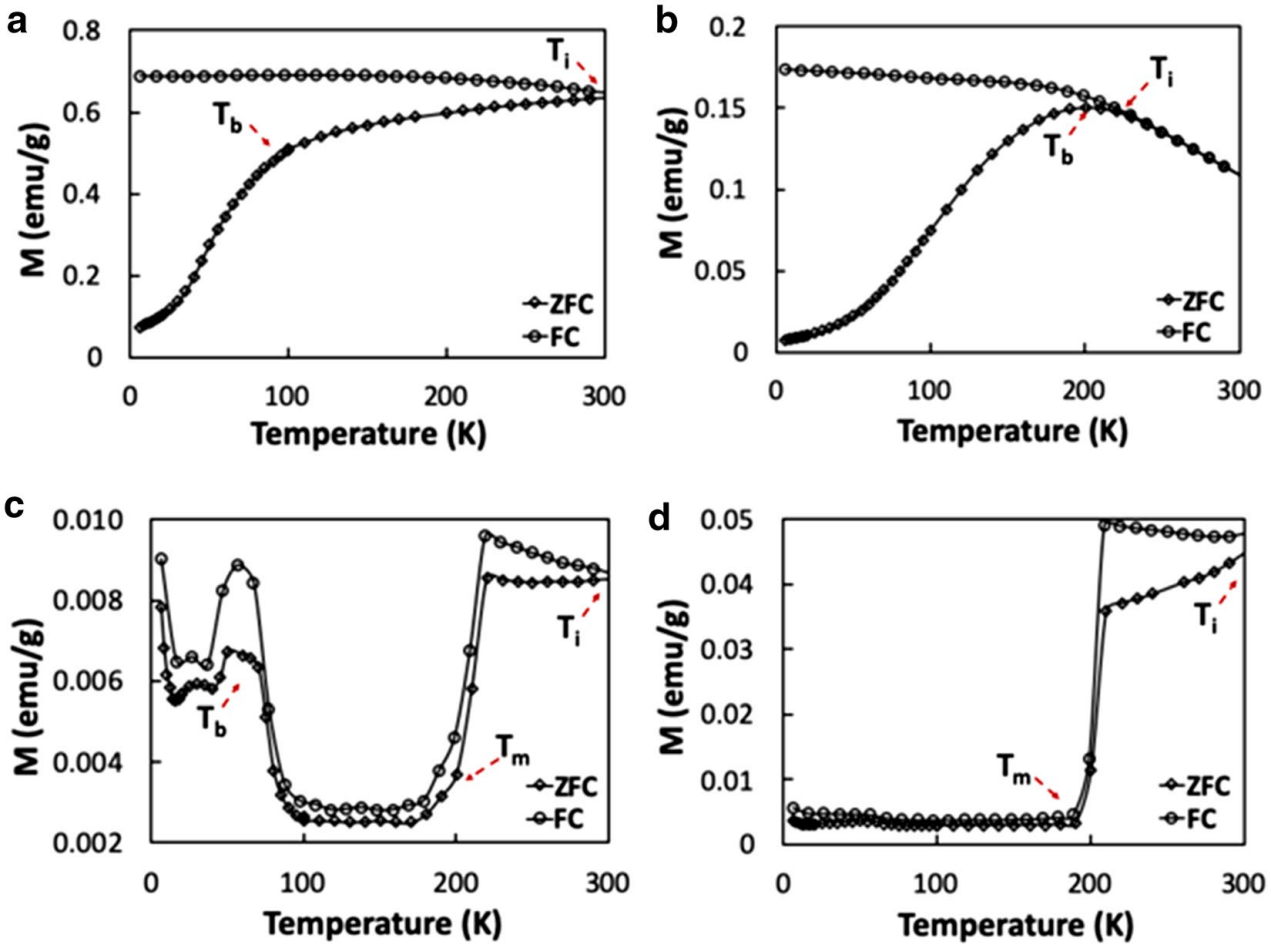
ZFC and FC curves at 100 Oe from 6 to 300 K displaying the Blocking Temperature (T_b_), Irreversibility Temperature (T_i_), and the Morin Temperature (T_m_) for the: **a** nanorods, **b** nanosheets, **c** nanodiamonds, **d** nanospheres

Zero-field cooling (ZFC) and field cooling (FC) curves were collected at 100 Oe from 6 K to 300 K for all the nano-sized hematite samples (Fig. 4). ZFC and FC curves were not collected for the rugby balls since they were magnetically similar to bulk hematite. In Fig. 4d, the ZFC and FC curves for the nanospheres mimicked those of bulk hematite; they showed irreversibility at temperatures higher than 300 K (*i.e.*, the curves do not overlap) due to particle-particle interactions [28]*.* The ZFC and FC curves of the nanodiamonds and the nanorods (Fig. 4c, a) also did not overlap, and they converge at room temperature (*i.e.*, irreversibility temperature at ~300 K; Table 2). On the other hand, the nanosheets show an irreversibility temperature of 229 K (Fig. 4b). The larger irreversibility temperatures of the nanosheets and nanorods, as compared to the other nanoparticles, indicated contributions of crystalline and shape anisotropy on their magnetic properties [29].

**Table 2:** ZFC and FC Curve related properties

Sample	Blocking Temperature (T_b_)	Irreversibility temperature (T_i_)	Morin temperature (T_m_)
nanorods	95 K	~ 300 K	–
nanosheets	201 K	229	–
nanodiamonds	57 K	~ 300 K	~ 209 K
nanospheres	–	> 300 K	~ 198 K

The Morin transition (T_m_)—the temperature where the magnetic properties of hematite transition from weakly ferromagnetic to antiferromagnetic—occurs at 198 K and 209 K for the nanospheres and nanodiamonds (Figs. 4c, d), respectively, indicating their weakly ferromagnetic nature at room temperature. Both Morin transition temperatures are less than that of bulk hematite (~263 K), caused by the small particle sizes of the nanospheres and nanodiamonds [11, 22, 30, 31]. Comparatively, the nanosheets and nanorods do not display a Morin transition since both samples have particles with sizes of 20 nm or less [31].

The nanodiamonds have a blocking temperature (below which the nanoparticles ceased to be superparamagnetic) of ~57 K (Fig. 4c). This material shows a paramagnetic Curie tail that suggests spin glass behavior at temperatures below 50 K [30, 32, 33]. The nanosheets and nanorods show blocking temperatures at 201 K and ~95 K, respectively, consistent with their superparamagnetic-like behavior.

### Effects crystallinity and shape on magnetism

The crystallinity of the samples was determined previously with SEM [23, 24] and compared to the calculated crystallite size of each nanomaterial—as determined from their respective XRD patterns and the Scherrer equation (Additional file 1: Table S1). From these images, polycrystalline materials were defined as particles having multiple grain boundaries that separated sections of difference crystal growth directions or crystallites. The grain boundaries serve as magnetic domains, which contributes to hysteretic heating of the material when subjected to an AMF [10]*.* Particles that did not present grain boundaries (i.e., having a crystallite size approximately equal to the average particle size determined by SEM) were concluded to be single crystalline particles. For sufficiently small enough particles, only a single crystallite exists within the particle volume, which results in a single magnetic domain, the absence of hysteresis, and the inability to undergo hysteretic heating [10].

The nanospheres, nanorods, and nanosheets were polycrystalline [23, 24], which is consistent with their observed magnetic hysteresis. They all reached magnetic saturation in fields less than 10 kOe, since fully rotating their magnetic domains into the direction of the magnetic field is a low energy process (i.e., domain wall motion) (Additional file 1: Figure S3, Table S2) [10]. The nanodiamonds were single crystalline. They did not reach magnetic saturation at a field strength of 50 kOe, as rotating the magnetic dipole of the single domain (i.e., domain rotation) is more energy intensive [10]. The rugby balls also required magnetic fields larger than 50 kOe to saturate due to their shape anisotropy [34].

Shape has a significant effect because elongating a nanoparticle increases its coercivity, magnetic remanence, and magnetic saturation values [10, 17, 18, 35–37], as seen in the high saturation values of the hematite nanosheets and nanorods (3.25 emu/g and 4.58 emu/g, respectively). The nanorods have a magnetic saturation value approx. 1.4× greater than nanosheets because magnetization decreases with increasing cross-sectional area (e.g., magnetization = pole strength / cross-sectional area) [10]. The surface spins (or the magnetic dipole moments of the surface atoms) presumably have a negligible effect on the magnetic properties because all the nanoparticle sizes are ≥ 4 nm [38–40].

#### Effects of shape, size, crystallinity & particle concentration on radio frequency (RF) heating

**Fig. 5:**
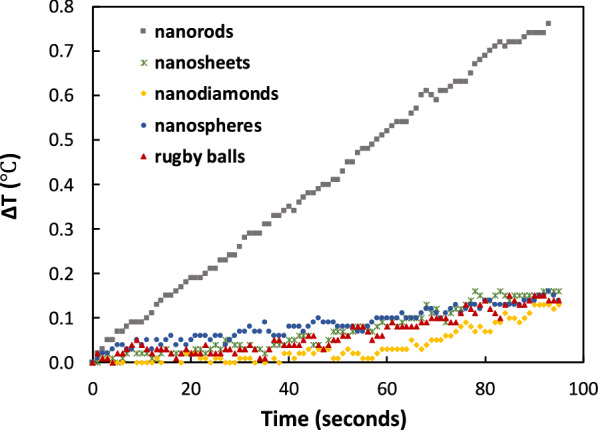
Temperature profiles for the hematite shapes (4 mg/mL). AMF heating conditions: field frequency = 325 kHz, field strength = 13.3 kA/m, average initial solution temperature = 23.9 ± 0.1 °C

In a typical AMF heating experiment, a water suspension was placed in a 2-mL cryovial and placed within Styrofoam insulation. This was then placed inside a 1 kW EASYHEAT induction heating system (Ambrell) and an AMF was applied for 90 seconds at a fixed frequency of 325 kHz (a common frequency used in literature, though not necessarily optimized for maximum heating for each hematite sample) [41]. Suspension temperatures were measured in real-time with a fiber optic temperature sensor. All hematite samples produced a linear and uniform increase in solution temperature over time (Fig. 5).

The nanorods show the fastest heating of the hematite samples, based on measured SAR values (Table 3, Additional file 1: Figure S4). SAR_meas_ was calculated using Equation 1 and from the slope of the experimentally determined temperature profiles of Fig. 5. We attribute this to their nanosize (i.e., <50 nm), elongated shape and superparamagnetic-like magnetic behavior. The nanorods generated heat via Brownian/Neel relaxations like that seen for superparamagnetic-like Fe_3_O_4_ nanoparticles (measured SAR values on the order of 50–200 W/g [41–43]) which have higher values of magnetic coercivity and saturation [44]. The nanorods are too small to generate heat via eddy current heating or hysteretic heating.

**Table 3:** Measured SAR values at two suspension concentrations

Sample	SAR_meas_ (W/g of α-Fe_2_O_3_) [4 mg/mL]	SAR_meas_ (W/g of α-Fe_2_O_3_) [1 mg/mL]
nanorods	43.5 ± 3.0	24.0 ± 7.0
nanosheets	4.13 ± 0.4	16.7 ± 4.4
nanodiamonds	3.15 ± 0.4	14.2 ± 3.8
nanospheres	6.88 ± 0.4	13.4 ± 1.7
rugby balls	5.18 ± 1.2	27.3 ± 3.4

The nanospheres had the next highest SAR_meas_ value (6.3× less, at a concentration of 4 mg/mL). More than twice as big, the rugby balls had a SAR_meas_ value that was within error of that of the nanospheres despite having the largest magnetic hysteresis loop (Fig. 3). Both of these shapes were too large for substantial Brownian/Neel heating but too small to undergo eddy current heating, suggesting that the temperature increased due to hysteretic heating. The rugby balls was expected to have a higher SAR value because magnetic heating is a function of coercivity and magnetic saturation [45]. More than likely, the selected frequency was not optimally tuned to the rugby ball’s particle size [25, 41].

The superparamagnetic-like nanosheets had the second lowest SAR_meas_ value despite having an enhanced magnetic saturation brought on by shape anisotropy. Although having nanometer thickness (4-7 nm), the nanosheets were ~200 nm wide, which is too large to undergo Brownian/Neel heating and too small for eddy current heating. Thus, they undergo hysteretic heating, albeit with a low SAR_meas_ value (due to their low coercivity and small hysteresis).

The nanodiamonds generated the lowest SAR_meas_ value of all the hematite samples (Table 3). Given their single crystalline nature, size (i.e., <50 nm) and very small hysteresis area (Fig. 3, inset), the superparamagnetic-like nanodiamonds generated heat via Brownian/Neel relaxations – like the nanorods – and not through eddy current heating or hysteretic heating. However, the nanodiamonds had a SAR_meas_ value ~14× lower than that of the nanorods, due to its very low magnetization measured at the maximum magnetic field (Table 1).

For the nanosheets, nanodiamonds, nanospheres, and rugby balls, the SAR_meas_ values increased when the particle concentration decreased from 4 mg/mL to 1 mg/mL (Table 3). In general, higher concentrations promote particle aggregation which lower SAR_meas_ [8, 14, 46]. This trend did not hold for the nanorods, however. At the lower concentration, the nanorods had a 1.8× lower SAR_meas_ value. Studies have shown that rod-like iron oxide nanoparticles align in the direction of the imposed magnetic field, which leads to larger SAR_meas_ values. Quite possibly, more nanorods are naturally aligned in the direction of the magnetic field at higher concentrations thus yielding the higher SAR_meas_ value.

## Conclusion

In conclusion, we found that increasing the axial anisotropy of hematite produced the largest influence on SAR_meas_ (i.e., 43.5 W/ g of α-Fe_2_O_3_ nanorods vs. 6.89 W/ g of α-Fe_2_O_3_ nanospheres). By exploring the relationship between hematite structure (i.e., size, shape, crystallinity) and its magnetic heating properties, we demonstrated that hematite nanorods can undergo radio frequency heating, generating appreciable SAR values that are 6 × greater than bulk hematite (i.e., rugby balls). They produce SAR_meas_ values close to those suggested for magnetic hyperthermia treatment of tumours (e.g., 50 W/g [16]). Such findings provide a footing for future studies into designing better hematite nanostructures for RF heating applications via understanding the structure–property relationships between the nanostructure, nano-magnetism, and magnetic heating.

## Supplementary Information

**Additional file 1.** Additional figures and tables.

## Data Availability

All data generated or analyzed during this study are included in this published article and its supplementary information files.
