# Supplementary material for: Nano-structural effects on Hematite (α-Fe2O3) nanoparticle radiofrequency heating
Source: Nano Converg. 2021 Mar 9;8:8. doi: 10.1186/s40580-021-00258-7 (PMC7940460; doi:10.1186/s40580-021-00258-7)
Supplement: Supplementary file 1 — Additional file 1. Additional figures and tables. [file 40580_2021_258_MOESM1_ESM.pdf]

Nano-structural effects on Hematite (α-Fe_2O_3) Nanoparticle Radiofrequency Heating

Supplemental Information

Camilah D. Powell, Amanda W. Lounsbury, Zachary S. Fishman, Christian L. Coonrod, Miranda J. Gallagher, Dino Villagran, Julie B. Zimmerman, Lisa D. Pfefferle, Michael S. Wong

Table S1: XRD Determined Crystallite Size

Sample Names	Avg. Size (nm)
nanorods	31
nanosheets	9
nanodiamonds	15
nanospheres	21
rugby balls	23

Table S2: Crystallinity of the Hematite Particles

Sample	Crystallinity
nanorods	Polycrystalline[2]
nanosheets	Polycrystalline[2]
nanodiamonds	Single crystal[1]
nanosphere	Polycrystalline[1]
rugby balls	Polycrystalline[1]

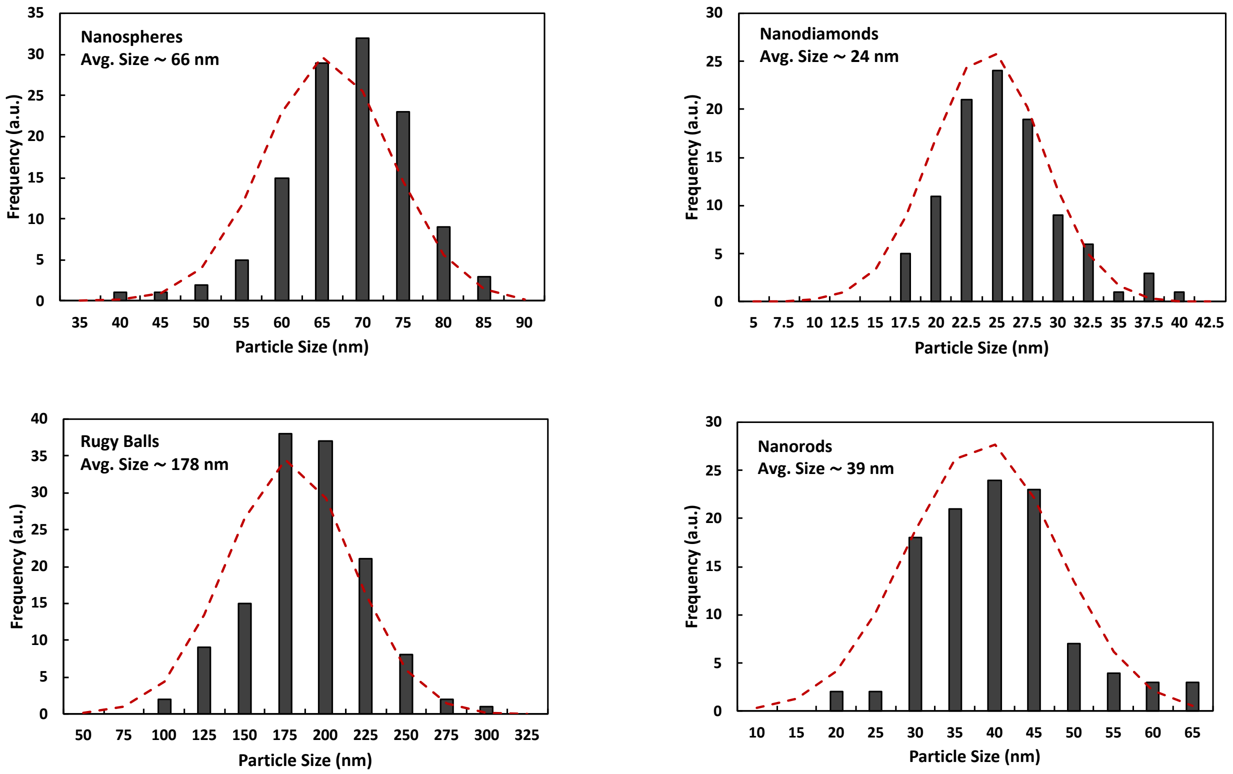

Figure S1: Particle size histograms of α-Fe_2O_3 nanomaterials.

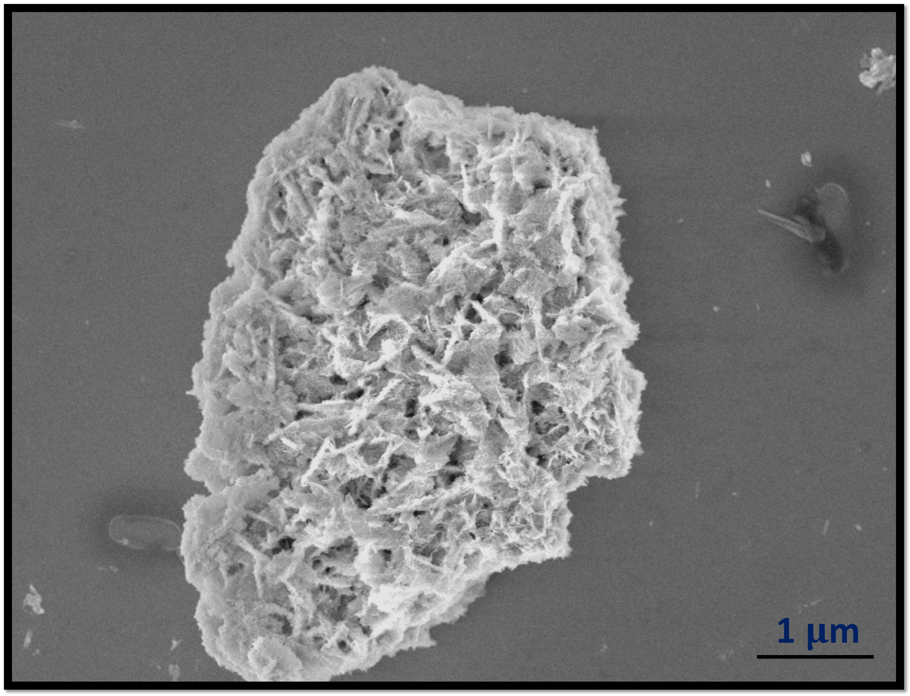

Figure S2: SEM image of the nanosheet morphology, scale bar: 1 µm.

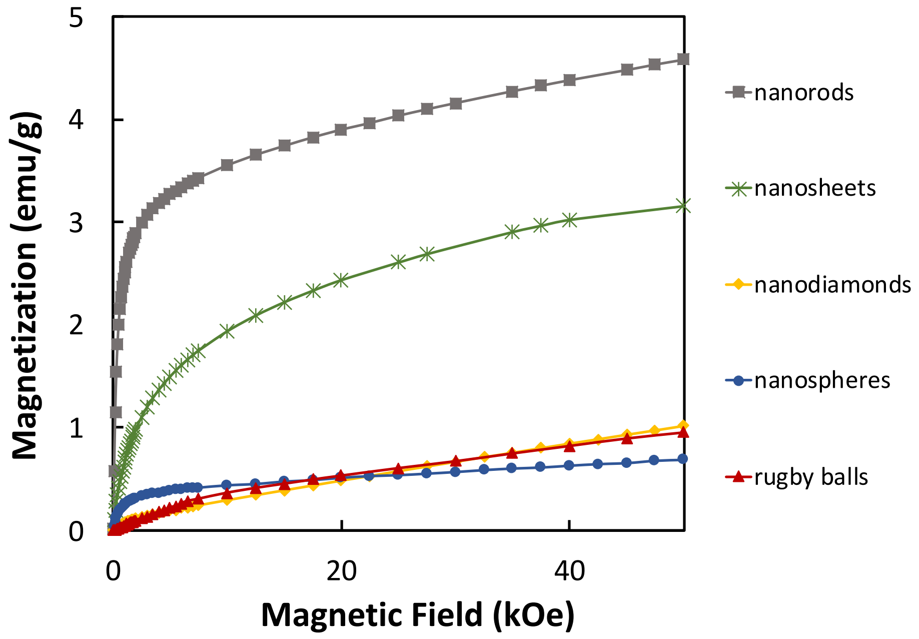

Figure S3: Magnetization curves at 300K from 0 kOe to 50 kOe for the hematite nanorods (grey squares), nanosheets (green asterisks), nanodiamonds (yellow diamonds), nanospheres (blue spheres), and rugby balls (red triangles) shaped particles.

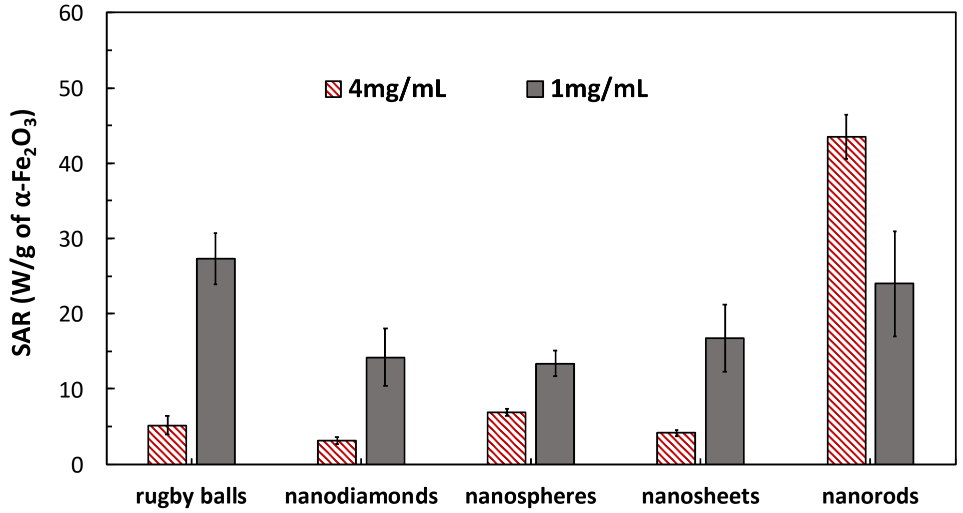

Figure S4: SAR values for the hematite solutions of varying concentrations; 4 mg/mL (red diagonal stripes) and 1 mg/mL (solid grey).

1 A. W. Lounsbury, R. Wang, D. L. Plata, N. Billmyer, C. Muhich, K. Kanie, T. Sugimoto, D. Peak and J. B. Zimmerman, *J. Colloid Interface Sci.*, 2019, **537**, 465–474.
2 Z. S. Fishman, Y. He, K. R. Yang, A. W. Lounsbury, J. Zhu, T. M. Tran, J. B. Zimmerman, V. S. Batista and L. D. Pfefferle, *Nanoscale*, 2017, **9**, 12984–12995.
3 S. Tong, C. A. Quinto, L. Zhang, P. Mohindra and G. Bao, *ACS Nano*, 2017, **11**, 6808–6816.
